# Prevalence of Moderate-to-Severe Premenstrual Syndrome or Premenstrual Dysphoric Disorder and Associated Variables Among Young Women in a Tertiary Health Care Center in Eastern India

**DOI:** 10.7759/cureus.69400

**Published:** 2024-09-14

**Authors:** Vineeta Vineeta, Vinayagamoorthy Venugopal, Priyanka Rai, Divyanshu Singh, Rashmi B Patel, Manish Raj

**Affiliations:** 1 Obstetrics and Gynaecology, All India Institute of Medical Sciences, Deoghar, Deoghar, IND; 2 Community and Family Medicine, All India Institute of Medical Sciences, Deoghar, Deoghar, IND; 3 Orthopaedics, All India Institute of Medical Sciences, Deoghar, Deoghar, IND

**Keywords:** premenstrual dysphoric disorder, premenstrual symptoms, premenstrual symptoms screening tool, quality of life, young females

## Abstract

Background: Premenstrual dysphoric disorder (PMDD) is a severe form of premenstrual syndrome (PMS) that is predominantly characterized by mood symptoms, and debilitating physical and cognitive symptoms, which have a direct or indirect impact on work efficiency, social and personal relationships, and overall quality of life. This study was conducted to determine the prevalence of moderate to severe PMS and PMDD among young women in India and investigate sociodemographic and other associated factors.

Materials and methods: We conducted a cross-sectional study that included 198 young women between 17 and 30 years of age who were medical students or nursing staff from a medical college in Eastern India. Data were collected regarding sociodemographic information, menstrual patterns, age of menarche, sexually active status, body mass index, and level of physical activity, which were analyzed to find correlations with moderate to severe PMS or PMDD. The Premenstrual Symptoms Screening Tool was used for diagnosing moderate to severe PMS or PMDD. The data were analyzed using Statistical Product and Service Solutions (SPSS, version 24.0; IBM SPSS Statistics for Windows, Armonk, NY) software.

Results: Forty-six (23.5%) respondents were diagnosed with moderate-to-severe PMS, of which four (2%) were diagnosed with PMDD. Among those diagnosed with moderate-to-severe PMS/PMDD, 39 (27.3%) were of the age group 17-24 years, and seven (13.2%) were of the age group 25-30 years, which was a statistically significant difference (p=0.05). Thirty-eight (30.6%) students were diagnosed with moderate-to-severe PMS, as compared to eight (11.1%) working professionals, which was also a statistically significant difference.

Conclusion: Moderate-to-severe PMS significantly impacts quality of life. Considering its prevalence among young subjects, it is important to make young girls aware of the condition and educate them regarding various ways to manage their symptoms.

## Introduction

Premenstrual syndrome (PMS) is quite common yet lacks considerable emphasis due to a lack of awareness and treatment-seeking among women, especially in India. PMS typically affects 20%-40% of women of reproductive age with a constellation of symptoms of varying severity [1]. PMS is characterized by the timing of symptoms, rather than symptom type, with onset typically one week before the beginning of menses (i.e., the luteal phase of the cycle), followed by symptom relief during menstruation. Physical symptoms of PMS include mastalgia, bloating, headaches, muscle and joint aches, and palpitations; psychological symptoms include irritability, low mood, anxiety, loss of interest in day-to-day activities, lack of concentration, and feelings of fatigue. Four out of 10 women (40%) suffer from PMS. Premenstrual dysphoric disorder (PMDD) is the more severe form of PMS with mood symptoms along with debilitating physical, cognitive, and affective symptoms that affect quality of life. In January 2022, PMDD was added to the 11th revision of the International Classification of Disease (ICD-11). According to the ICD-11, PMDD is diagnosed if the symptoms occur in the luteal phase in most cycles within the past year (12 months), which must include affective symptoms and cognitive or somatic symptoms, and the condition must cause significant distress [2]. PMDD has a global prevalence of approximately 2%-8% [3]. A systematic review and meta-analysis estimated the prevalence of PMDD in the Indian population to be 3.7%-65.7%, attributed to sociodemographic and cultural factors, variations in diagnostic tools, and the lack of large population-based studies [1].

Despite PMS/PMDD affecting a significant percentage of women of reproductive age at some point in their lives, the exact pathophysiology remains unclear. It is known that PMS/PMDD is triggered by the progesterone hormone and its metabolites allopregnanolone and pregnanolone, which are potent neuroactive steroids. These progesterone metabolites bind to the neurosteroid-binding sites of gamma-aminobutyric acid (GABA) receptors and change their configuration, making them resistant to further activation, leading to decreased central mediated GABA inhibition [4]. Lowered levels of serotonin have been associated with PMS-like symptoms, with drugs that increase serotonin levels (e.g., selective serotonergic reuptake inhibitors) efficacious in PMS/PMDD even when given during the luteal phase [5]. Menstrual abnormalities (e.g., dysmenorrhea), obesity, and lifestyle factors (e.g., lack of sleep, stress, low levels of physical activity, consumption of caffeine, junk foods, and high levels of salt) have inconsistently been associated with PMS [6].

This study aimed to evaluate the prevalence of moderate to severe PMS/PMDD and their related factors among a young female population in a tertiary health care center.

## Materials and methods

We performed a cross-sectional descriptive-analytical study at an institute of national importance in the eastern region of India. 

Sample size calculation: Based on previous studies, the prevalence of PMS and PMDD is approximately 50%. With a 95% confidence interval and 5% margin of error, for a population of 400 young female medical students and nursing staff who were residing in the college during the study period, the sample size was calculated to be 196 according to the following formula: 

$CI'=\hat{p} \pm \text{z} \times \sqrt{\frac{\hat{p}(1-\hat{p})}{n'}\times \frac{N-n'}{N-1}}$

$\text{where}\\ \text{z is the z score}\\ \hat{p} \text{ is the population proportion}\\ n' \text{ is sample size}\\ N \text{ is the population size}$

After obtaining ethical clearance from the Institutional Ethical Committee of the college (approval number: 2021-13-IND-02), female medical students and staff working in the institute were invited to participate in the study by completing Google Forms. We conducted 15-20-minute sessions to explain the purpose and the inclusion and exclusion criteria of the study to the participants and ensured them that their anonymity would be maintained. The participants provided written informed consent before being included in the study. Data collection was done with a two-part, self-administered questionnaire. The first part of the questionnaire comprised socio-demographic information (age, sexual activity status, urban/rural background), chronic medical illness, personal history of psychiatric illness, family history of psychiatric illness, substance abuse, menstrual abnormalities, age of menarche, body mass index (BMI), and daily physical activity status. The second part of the questionnaire comprised the Premenstrual Symptoms Screening Tool (PSST) created by Steiner et al. [7], which also had two parts. The first part included a list of 14 premenstrual symptoms (covering mood, behavioral, and physical dimensions), which were scored on a 4-point Likert scale as “not at all,” “mild,” “moderate,” or “severe” based on their experience during menstrual cycles. The second part of the PSST comprised five questions to measure the impact of these symptoms on their day-to-day life. The participants scored their symptoms based on their experience during most menstrual cycles in the last 12 months. Based on the above scale, the participants were categorized as PMDD, moderate to severe PMS, or mild/no PMS [7].

Using the PSST, PMDD was diagnosed when the following criteria were met: in the first part of the questionnaire, one of items 1-4 was rated as severe; at least four of items 1-14 were rated as moderate to severe; and, in the second part, one item was rated as severe. Similarly, moderate-to-severe PMS was diagnosed when, in the first part, one of items 1-4 was rated as moderate to severe; at least four of items 1-14 were rated as moderate to severe; and, in the second part, at least one item was rated as moderate to severe. The PSST was used because of its good reliability, with an acceptable Cronbach’s alpha value of 0.90 [8].

All female medical and paramedical students and nursing staff working in the tertiary health care center who gave written consent were included in the study. 

Patients with known psychosis or on anti-psychotic medication and those with primary amenorrhea were excluded from the study. The data were analyzed with Statistical Product and Service Solutions (SPSS, version 24.0; IBM SPSS Statistics for Windows, Armonk, NY) SPSS software. The chi-squared test was used to compare qualitative variables. The analysis of variance test with post-hoc analysis (Kruskal-Wallis test) was used to compare the differences between means of respective groups (i.e., no/mild PMS vs. moderate to severe PMS/PMDD), with a p-value of <0.05 indicating statistical significance.

## Results

As seen in Table 1, 46 (23.4%) respondents were diagnosed with moderate-to-severe PMS, of which four (2%) were diagnosed with PMDD. Regarding the baseline characteristics of our 196 respondents, 150 (76.5%) were 17-24 years of age, and 46 (23.5%) were 25-30 years of age. Seventy (35.7%) were of a rural background, and 126 (64.3%) were of an urban background. A total of 169 (86.2%) were sexually inactive, as compared to 27 (13.8%) who were sexually active; 124 (63.3%) were students, whereas 72 (36.7%) were working professionals. Among those diagnosed with moderate-to-severe PMS, 39 (27.3%) were 17-24 years of age, and seven (13.2%) were 25-30 years of age, and this difference was statistically significant (p = 0.05). No difference was observed in the PMS diagnosis rate with regards to urban/rural or religious background, as well as history of sexual activity. Thirty-eight (30.6%) students were diagnosed as suffering from moderate-to-severe PMS, as compared to 8 (11.1%) working professionals, and this difference was statistically significant (p = 0.02). No significant difference was found regarding family history or personal history of psychiatric illness or chronic medical illness among either group. Moderate-to-severe PMS/PMDD was found not to be associated with the age of menarche, length of cycle, pain during a cycle, higher BMI, or level of physical activity (Table 2).

**Table 1:** Prevalence of PMS/PMDD. PMDD: Premenstrual dysphoric disorder; PMS: premenstrual syndrome

Category	Frequency (n)	Percentage (%)
No/Mild PMS	150	76.6
Moderate to Severe PMS	46	23.4
Moderate to severe PMS with PMDD	4	2

**Table 2:** Comparison of socio-demographic, menstrual cycle, age of menarche, BMI, and daily physical activities amongst respondents. ^ Column percentage, # Row percentage; a p-value of 0.05 is statistically significant. BMI-Body mass index; PCOS: Polycystic ovarian syndrome

Sl. No.	Characteristics	Total		Moderate-to-Severe PMS/PMDD		No/Mild PMS		Significance Kruskal-Walis test/ꭓ2 (p-value)
		n	(%^^^)	n	(%^#^)	N	(%^#^)	
1	Age							
	17-24	150	(76.5)	39	(27.3)	104	(72.7)	0.04*
	25-30	46	(23.5)	07	(13.2)	46	(86.8)	
2	Residence							
	Rural	70	(35.7)	15	(21.4)	55	(78.6)	0.61
	Urban	126	(64.3)	31	(24.6)	95	(75.4)	
3	Religion							
	Hindu	171	(87.2)	37	(21.6)	134	(78.4)	
	Muslim	12	(6.1)	6	(50)	6	(50)	0.08
	Others	13	(6.6)	3	(23.1)	10	(76.9)	
4	Sexual History							
	Active	27	(13.8)	4	(14.8)	23	(85.2)	0.25
	Inactive	169	(86.2)	42	(24.9)	127	(75.1)	
5	Current Profession							
	Student	124	(63.3)	38	(30.6)	86	(69.4)	0.02*
	Working	72	(36.7)	8	(11.1)	64	(88.9)	
6	Family History of Psychiatric Illness							
	Present	13	(6.6)	5	(38.5)	8	(61.5)	
	Absent	183	(93.4)	41	(22.4)	142	(77.6)	
7	Personal Psychiatric Illness							
	Present	6	(3.1)	1	(16.7)	5	(83.3)	0.69
	Absent	190	(96.9)	45	(23.7)	145	(76.3)	
8	History of chronic medical illness							
	Present	23	(11.7)	3	(13)	20	(87)	0.21
	Absent	173	(88.3)	43	(24.9)	130	(75.1)	
9	Substance Abuse							
	Present	2	(1)	0	(0)	2	(100)	0.43
	Absent	194	(99)	46	(23.7)	148	(76.3)	
10	Age of Menarche							
	<10 years	7	(3.6)	2	(28.6)	5	(71.4)	0.84
	11-14 years	168	(85.7)	40	(23.8)	128	(76.2)	
	>15 years	21	(10.7)	4	(19)	17	(81)	
11	Length of Menstrual Cycle							
	<24 days	1	(.5)	1	(100)	0	(0)	0.12
	24-38 days	187	(95.4)	42	(22.5)	145	(77.5)	
	>38 days	8	(4.1)	3	(37.5)	5	(62.5)	
12	Pain During Menstruation							
	No	23	(11.7)	3	(13)	20	(87)	
	Mild	72	(36.7)	12	(16.7)	60	(83.3)	
	Moderate	82	(41.8)	24	(29.3)	58	(70.7)	
	Severe	19	(9.7)	7	(36.8)	12	(63.2)	
13	Gynaecological Illness							
	No	170	(86.7)	38	(22.4)	132	(77.6)	0.39
	PCOS	22	(11.2)	6	(27.3)	16	(72.7)	
	Others	4	(2)	2	(50)	2	(50)	
14	Daily Physical Exercise							
	Yes	69	(35.2)	15	(21.7)	54	(78.3)	0.67
	No	127	(64.8)	31	(24.4)	96	(75.6)	
15	BMI in kg/m^2^							
	<18.5	29	(14.8)	3	(10.3)	26	(89.7)	0.223
	18.5-24.9	99	(50.5)	23	(23.2)	76	(76.8)	
	25-29.9	37	(18.9)	10	(27)	27	(27)	
	>30	31	(15.8)	10	(32.3)	21	(21)	

As seen in Table 3, among all physical or mood symptoms, anger/irritability, depressed mood, and lack of energy/fatigue symptoms were found in all respondents with moderate to severe PMS/PMDD. Anger/irritability (80.4%) was the most common and highest-intensity symptom (moderate-to-severe category) experienced by respondents with moderate-to-severe PMS (n = 43). Hypersomnia was more common (n = 39; 84.7%) than insomnia (n = 25; 54.3%) among the respondents. Thirty-eight (82.6%) respondents reported that their work efficiency/productivity was significantly affected by their symptoms, with 100% of those suffering from moderate to severe PMS/PMDD (n = 46) being affected to some extent. The effect of PMS/PMDD on the quality of life of the respondents is summarized in Table 4. Regarding the treatment-seeking behavior of those diagnosed with moderate-to-severe PMS, only four (8%) respondents received treatment to combat their symptoms despite being significantly affected by the condition.

**Table 3:** Presence of symptoms among those with moderate-to-severe PMS (N=46).

Symptom	Severe % (n)	Moderate % (n)	Mild % (n)	Not at all % (n)
Anger/irritability	6.5 (9)	73.9 (34)	19.6 (3)	-
Anxiety/tension	10.9 (5)	60.9 (28)	23.9 (11)	4.3 (2)
Tearfulness/increased sensitivity to rejection	13 (6)	58.7 (27)	19.6 (9)	8.7 (4)
Depressed mood/helplessness	13 (6)	56.5 (26)	30.4 (14)	-
Decreased interest in home activities	13 (6)	50 (23)	32.6 (15)	4.3 (2)
Decreased interest in work activities	10.9 (5)	47.8 (22)	30.4 (14)	8.7 (5)
Decreased interest in social activities	13 (6)	47.8 (22)	23.9 (11)	15.2 (7)
Difficulty concentrating	10.9 (5)	50 (23)	23.9 (11)	15.2 (7)
Lack of energy/fatigue	13 (6)	67.4 (31)	19.6 (9)	-
Overheating/food cravings	6.5 (3)	30.4 (14)	30.4 (14)	32.6 (15)
Insomnia/inability to sleep	4.3 (2)	19.6 (9)	30.4 (14)	45.7 (21)
Hypersomnia/need for more sleep	6.5 (3)	54.3 (25)	23.9 (11)	15.2 (7)
Feeling overwhelmed/out of control	6.5 (3)	28.3 (13)	30.4 (14)	34.8 (16)
Physical symptoms of breast tenderness/muscle pain/joint pain	10.9 (5)	45.7 (21)	28.3 (13)	15.2 (7)

**Table 4:** Effect of PMS/PMDD on quality of life of respondents. PMDD: Premenstrual dysphoric disorder; PMS: premenstrual syndrome

Nature of condition	Severe % (n)	Moderate % (n)	Mild % (n)	Not at all % (n)
Work efficiency	4.3 (2)	78.3 (36)	17.4 (8)	0 (0)
Relationship with family	13 (6)	45.7 (21)	30.4 (14)	10.9 (5)
Social responsibilities	2.2 (1)	43.5 (20)	43.5 (20)	10.9 (5)
Home responsibilities	4.3 (2)	41.3 (19)	41.3 (19)	13 (6)

## Discussion

Frequency of moderate-to-severe PMS/PMDD 

The current study aimed to estimate the prevalence of moderate-to-severe PMS and PMDD among young medical professionals aged 17-30 years. In this study, the prevalence of moderate-to-severe PMS and PMDD was 23.4% and 2%, respectively. Previous studies have reported a prevalence of moderate to severe PMS and PMDD of 25.1%-49.8% and 4.3%-44.5%, respectively [9-14]. In a systematic review and meta-analysis by Dutta et al. of 25 Indian studies, the pooled prevalence of PMS and PMDD was 43% (95% CI: 0.35-0.5) and 8% (95% CI: 0.6-1.0), respectively [1]; however, they included all cases of PMS contrary to our study, which included only those with moderate-to-severe PMS. This difference may explain the wide variation in the results. According to global data, the prevalence of PMS and PMDD is 20%-40% and 2%-8%, respectively [1,15].

Age, urban/rural background, sexually active/inactive, menstrual patterns, and age of menarche 

In our study, the prevalence of PMS/PMDD was higher among younger subjects aged 17-24 years (n=39; 27.3%) compared to older subjects aged 25-30 years (n=7; 13.2%), which was found to be a statistically significant difference. The prevalence of PMS/PMDD was found to be higher in medical students compared to working medical professionals, which was a statistically significant difference. This observation aligned with other studies that found a higher prevalence of severe PMS/PMDD among younger populations, which suggested policies and interventions targeting adolescent individuals [16,17]. No difference in occurrence was found based on urban vs. rural background, sexual activity, age of menarche, or menstrual pattern, with similar results found in other studies [6,3,15]. Dutta et al. found no correlation between PMS/PMDD and menstrual pattern, dysmenorrhea, or place of residence [1]. Pooled odds ratios of various studies in a meta-analysis of PMS in Ethiopian women by Geta et al. showed no significant correlation between the early age of menarche and the likelihood of PMS [17]. Many previous studies showed an association between menstrual pain and moderate to severe PMS and PMDD, which differs from our study, as no significant association could be established [18,19]. Some studies have compared rural and illiterate with urban and literate populations, with PMS being more common among urban and literate individuals, which was attributed to increased levels of perception and reporting [20,21]. However, in this study, as literacy was almost comparable among all respondents, background (i.e., rural vs. urban) did not have a significant effect on the results, suggesting little if any influence of environmental factors as a causal factor for PMS/PMDD.

BMI and physical activity 

In line with other studies, no association was found between PMS/PMDD, and BMI and high levels of physical activity [15,17,22-24]. However, in contrast, Sahin et al. found that the prevalence of PMS was 8.2% higher in obese patients [25]. Similarly, in a prospective epidemiological study on nurses over a 10-year period by Bertone-Johnson et al., there was a strong linear association between BMI at baseline and risk of PMS, with every 1 kg/m^2^ increase in BMI associated with a 3% increase in PMS risk [26].

Physical and psychological symptoms 

Anger/irritability was found to be the most common symptom of moderate-to-severe intensity, affecting 80.4% of respondents diagnosed with moderate-to-severe PMS/PMDD, similar to other studies [15,27-30]. All respondents with this symptom reported that it had a significant effect on their work efficiency. Depressed mood was also present in all respondents diagnosed with PMS/PMDD. Despite all those with moderate-to-severe PMS/PMDD reporting some effect on work efficiency during menstrual cycles, only 8% had sought treatment for the same. Dismissal of these symptoms as routine in and around the time of menses, especially by parents and peers, may explain this behavior. Lack of knowledge regarding the condition and availability of treatment options can act as barriers to self-reporting by these women. Developing countries such as India are still characterized as being socially conservative with myths and taboos surrounding menstrual cycles; much work remains in educating and increasing awareness regarding these issues, especially among young women.

Summary

This study showed that the prevalence of moderate-to-severe PMS/PMDD amongst the young medical population is similar to global data. A significant relationship was found between age and moderate-to-severe PMS/PMDD. Anger/irritability was the most common symptom. Their treatment-seeking behavior is poor despite their living on the hospital campus highlights the ignorance amongst them regarding this condition. The PSST tool is an easy-to-use tool to screen for the condition. Awareness campaigns targeting adolescent girls such as senior secondary school and college girls need to be done. Prospective larger population-based studies will help explore this condition better in order to have a better understanding.

## Conclusions

Moderate-to-severe PMS has a significant impact on quality of life. With a higher prevalence among younger populations, it is important to make young women aware of the condition and to educate them regarding various ways to manage their symptoms. Apart from pharmacological measures, nonpharmacological interventions in the form of yoga, mindfulness exercises, and breathing exercises can be tried for treatment. The lack of large population-based studies and variations in studies regarding causative factors warrant further community-based research.

## Appendices

**Table 5:** The premenstrual symptoms screening tool (PSST). Do you experience some or any of the following premenstrual symptoms that start before your period and stop within a few days of bleeding? (Please mark an "X" in the appropriate box)

Symptom	Not at all	Mild	Moderate	Severe
1. Anger/irritability				
2. Anxiety/tension				
3. Tearful Increased sensitivity to rejection				
4. Depressed mood/hopelessness				
5 Decreased interest in work activities				
6. Decreased interest in home activities				
7. Decreased interest in social activities				
8. Difficulty concentrating				
9. Fatigue/lack of energy				
10. Overeating/food cravings				
11. Insomnia				
12. Hypersomnia (needing more sleep)				
13. Feeling overwhelmed or out of control				
14. Physical symptoms: breast tenderness, headaches, Joint/muscle pain, bloating, weight gain				

**Table 6:** Have your symptoms, as listed above, interfered with.

	Not at all	Mild	Moderate	Severe
You work efficiency or productivity				
Your relationships with coworkers				
Your relationships with your family				
Your social life activities				
Your home resposibllitles				

Scoring

The following criteria must be present for a diagnosis of PMDD:

1) At least one of #1, #2, #3, #4 is severe

2) In addition at least four of #1-#14 are moderate to severe

3) At least one of A, B, C, D, E is severe

The following criteria must be present for a diagnosis of moderate to severe PMS:

1) At least one of #1, #2, #3, and #4 is moderate to severe

2) In addition at least four of #1-#14 are moderate to severe

3) At least one of A, B, C, D, E is moderate to severe

Reference: [7]

## References

[1] Dutta A, Sharma A (2021). Prevalence of premenstrual syndrome and premenstrual dysphoric disorder in India: a systematic review and meta-analysis. Health Promot Perspect.

[2] American Psychiatric Association (2013). Diagnostic and Statistical Manual of Mental Disorders. Diagnostic and Statistical Manual of Mental Disorders, Fifth Edition.

[3] Gao M, Zhang H, Gao Z, Cheng X, Sun Y, Qiao M, Gao D (2022). Global and regional prevalence and burden for premenstrual syndrome and premenstrual dysphoric disorder: a study protocol for systematic review and meta-analysis. Medicine (Baltimore).

[4] Rapkin AJ, Akopians AL (2012). Pathophysiology of premenstrual syndrome and premenstrual dysphoric disorder. Menopause Int.

[5] Marjoribanks J, Brown J, O'Brien PM, Wyatt K (2013). Selective serotonin reuptake inhibitors for premenstrual syndrome. Cochrane Database Syst Rev.

[6] Babapour F, Elyasi F, Shahhosseini Z, Hosseini Tabaghdehi M (2023). The prevalence of moderate-severe premenstrual syndrome and premenstrual dysphoric disorder and the related factors in high school students: a cross‐sectional study. Neuropsychopharmacol Rep.

[7] Steiner M, Macdougall M, Brown E (2003). The premenstrual symptoms screening tool (PSST) for clinicians. Arch Womens Ment Health.

[8] Hariri FZ, Moghaddam-Banaem L, Siah Bazi S, Saki Malehi A, Montazeri A (2013). The Iranian version of the premenstrual symptoms screening tool (PSST): a validation study. Arch Womens Ment Health.

[9] Farrokh-Eslamlou H, Oshnouei S, Heshmatian B, Akbari E (2015). Premenstrual syndrome and quality of life in Iranian medical students. Sex Reprod Healthc.

[10] SeyedTabaee S R, Rahmatinejad P, Feridooni K (2019). Prevalence and severity of premenstrual syndrome and its relationship with psychological well-being in students of Qom University of Medical Sciences, (Iran). Qom Univ Med Sci J.

[11] Ahmadi F, Naghizadeh MM, Direkvand-Moghadam A, Mohamadian F, Ghazanfari Z (2018). A study on premenstrual syndromes of high school girl-students in Ilam City (Western Iran), 2015. J Ilam Univ Med Sci.

[12] Alavi A, Salahi-Moghaddam AR, Alimalayeri N, Ramezanpour A (2007). Prevalence of clinical manifestations of premenstrual syndrome and premenstrual dysphoric disorder in students of Bandar Abbas Medical University (in Persian). HMJ.

[13] Tatari F, Shaker J, Hosseini M, Rezaii M, Amirian M, Amirian F (2007). [Frequency of pre-menstrual dysphoric disorder (PMDD), premenstrual syndrome (PMS) and some related factors in students of girls' high schools of Kermanshah]. RBS.

[14] Farhadi Nasab A, Mani Kashani (2006). Study the prevalence of premenstrual dysphoric disorder in Hamadan high school girls in 2003. J Clin Med.

[15] Durairaj A, Ramamurthi R (2019). Prevalence, pattern and predictors of premenstrual syndrome (PMS) and premenstrual dysphoric disorder (PMDD) among college girls. New Indian J OBGYN.

[16] Kroll-Desrosiers AR, Ronnenberg AG, Zagarins SE, Houghton SC, Takashima-Uebelhoer BB, Bertone-Johnson ER (2017). Recreational physical activity and premenstrual syndrome in young adult women: a cross-sectional study. PLoS One.

[17] Geta TG, Woldeamanuel GG, Dassa TT (2020). Prevalence and associated factors of premenstrual syndrome among women of the reproductive age group in Ethiopia: systematic review and meta-analysis. PloS one.

[18] Raval CM, Panchal BN, Tiwari DS, Vala AU, Bhatt RB (2016). Prevalence of premenstrual syndrome and premenstrual dysphoric disorder among college students of Bhavnagar, Gujarat. Indian J Psychiatry.

[19] Bhuvaneswari K, Rabindran P, Bharadwaj B (2019). Prevalence of premenstrual syndrome and its impact on quality of life among selected college students in Puducherry. Natl Med J India.

[20] Marván ML, Díaz-Erosa M, Montesinos A (1998). Premenstrual symptoms in Mexican women with different educational levels. J Psychol.

[21] Cenac A, Maikibi DK, Develoux M (1987). Premenstrual syndrome in Sahelian Africa. A comparative study of 400 literate and illiterate women in Niger. Trans R Soc Trop Med Hyg.

[22] Rad M, Sabzevary MT, Dehnavi ZM (2018). Factors associated with premenstrual syndrome in female high school students. J Educ Health Promot.

[23] Masho SW, Adera T, South-Paul J (2005). Obesity as a risk factor for premenstrual syndrome. J Psychosom Obstet Gynaecol.

[24] Mishra A, Banwari G, Yadav P (2015). Premenstrual dysphoric disorder in medical students residing in hostel and its association with lifestyle factors. Ind Psychiatry J.

[25] Sahin S, Ozdemir K, Unsal A (2014). Evaluation of premenstrual syndrome and quality of life in university students. J Pak Med Assoc.

[26] Bertone-Johnson ER, Hankinson SE, Willett WC, Johnson SR, Manson JE (2010). Adiposity and the development of premenstrual syndrome. J Womens Health (Larchmt).

[27] Bakhshani NM, Mousavi MN, Khodabandeh G (2009). Prevalence and severity of premenstrual symptoms among Iranian female university students. J Pak Med Assoc.

[28] Steiner M, Peer M, Palova E, Freeman EW, Macdougall M, Soares CN (2011). The premenstrual symptoms screening tool revised for adolescents (PSST-A): prevalence of severe PMS and premenstrual dysphoric disorder in adolescents. Arch Womens Ment Health.

[29] Tabassum S, Afridi B, Aman Z, Tabassum W, Durrani R (2005). Premenstrual syndrome: frequency and severity in young college girls. J Pak Med Assoc.

[30] Pearlstein T, Yonkers KA, Fayyad R, Gillespie JA (2005). Pretreatment pattern of symptom expression in premenstrual dysphoric disorder. J Affect Disord.

